# Secondary Metabolites from Food-Derived Yeasts Inhibit Virulence of Candida albicans

**DOI:** 10.1128/mBio.01891-21

**Published:** 2021-08-17

**Authors:** Lohith Kunyeit, Nawneet K. Kurrey, K. A. Anu-Appaiah, Reeta P. Rao

**Affiliations:** a Department of Microbiology and Fermentation Technology, CSIR-Central Food Technological Research Institute (CFTRI), Mysuru, India; b Academy of Scientific and Innovative Research (AcSIR), Ghaziabad, India; c Department of Biology and Biotechnology, Worcester Polytechnic Institute, Worcester, Massachusetts, USA; d Department of Biochemistry, CSIR-Central Food Technological Research Institute (CFTRI), Mysuru, India; Tel Aviv University

**Keywords:** beneficial microbes, aromatic alcohols, *Candida albicans* biofilm, adhesion, Caco-2 cell monolayer, *Caenorhabditis elegans*, *Candida albicans*, food-derived yeast, phenylethanol, plastic adhesion, probiotic yeast, tryptophol

## Abstract

A sparse number of available antifungal drugs, therapeutic side effects, and drug resistance are major challenges in current antifungal therapy to treat Candida albicans-associated infections. Here, we describe two food-derived yeasts, Saccharomyces cerevisiae and Issatchenkia occidentalis, that inhibit virulence traits of C. albicans, including hyphal morphogenesis, biofilm formation, and adhesion to intestinal epithelial cells. These yeasts also protect the model host Caenorhabditis elegans from C. albicans infection. We demonstrate that the protective activity is primarily retained in the secretome of the beneficial yeasts, and the protection they provide as a physical barrier is negligible. S. cerevisiae *aro8 aro9* mutant analysis demonstrate that phenylethanol and tryptophol are necessary for protection, and experiments with commercially procured compounds indicate that they are sufficient to inhibit C. albicans virulence. We propose food-derived yeasts as an alternative or combination therapy to conventional antifungal therapy for C. albicans infection.

## INTRODUCTION

The polymorphic yeast Candida albicans has been associated with a range of infection outcomes from superficial ones, such as thrush and vaginitis, to life-threatening invasive bloodstream infections. A multilateral interaction with the host immune system and host microbiota maintains this opportunistic pathogen in a commensal form in the gastrointestinal and urogenital tracts (1). However, a condition of immune suppression or disruption of the host microbiota can cause severe mortality and morbidity (2). There are a limited number of drugs used to control *Candida* infection, most of which exhibit unpleasant side effects. Frequent usage of antifungal drugs drives resistance due to mutations and overexpression of drug efflux pumps and has posed a major clinical challenge (3). Furthermore, the complex structure of biofilms, which harbor a community of microorganisms, facilitates resistance to antifungals (4, 5). These challenges warrant alternative solutions for *Candida* infections.

In mixed microbial communities, secondary metabolites produced by one species have been shown to modulate virulence traits in other microbes (6). These secondary metabolites serve as environmental sensors that may respond to cell density and regulate key determinants of several microbial properties such as morphological transition and biofilm development. Secondary metabolites such as tyrosol, phenylethanol, and tryptophol have been shown to regulate filamentation and biofilm formation and/or inhibition in yeasts such as C. albicans, Saccharomyces cerevisiae, and Kloeckera apiculata (7–9). Some aromatic alcohols are mediated by cell density, while others have been shown to antagonize microbes in mixed culture. For example, *in vitro* studies demonstrate that phenylethanol triggers adhesion and biofilm formation in K. apiculata (9). S. cerevisiae produces phenylethanol and tryptophol which autoinduce pseudohyphal growth (7). C. albicans also produces these aromatic alcohols (10), but they are not involved in morphological transition. Together, these studies suggest species-specific roles for these aromatic alcohols. While these disparate *in vitro* studies suggest a role as biotherapeutic agents for secondary metabolites derived from beneficial yeasts, there is limited evidence *in vivo*.

More recently, consortia of bacteria have been used in clinical applications to modify the gut microbiome to treat and prevent bowel diseases. Yeasts have not yet been approved for therapeutic applications even though several studies have shown that yeasts such as Saccharomyces cerevisiae var. *boulardii* (S. boulardii) and S. cerevisiae prevent colonization and virulence of C. albicans (11–13). We and others have previously reported that beneficial yeasts inhibit virulence traits such as adhesion and biofilm development of many pathogenic fungi (14, 15). However, the molecular mechanism of the inhibitory activity has not yet been elucidated. Beneficial resident microbiota are an important component of innate immunity and are generally thought to compete for limited resources in the host environment. However, appropriate host infection model systems have been a limiting factor in such studies. Here, we demonstrate that food-derived yeasts pose a physical barrier and, more importantly, secrete aromatic alcohols that inhibit biofilm and pathogen adhesion under *in vitro* and *ex vivo* conditions. Furthermore, we used the model host Caenorhabditis elegans to demonstrate that these secondary metabolites are necessary and sufficient to protect the host from C. albicans infection. Purified forms of these aromatic alcohols inhibited C. albicans hyphal morphogenesis and biofilm formation at concentrations of ≥100 μM and protected C. elegans from C. albicans infection. Because the rise of drug-resistant fungal infections undermines the limited repertoire of treatment options, we propose that beneficial yeasts could be used to control and prevent *Candida* infection.

## RESULTS

### Food-derived yeasts inhibit virulence traits of Candida albicans.

**FIG 1:**
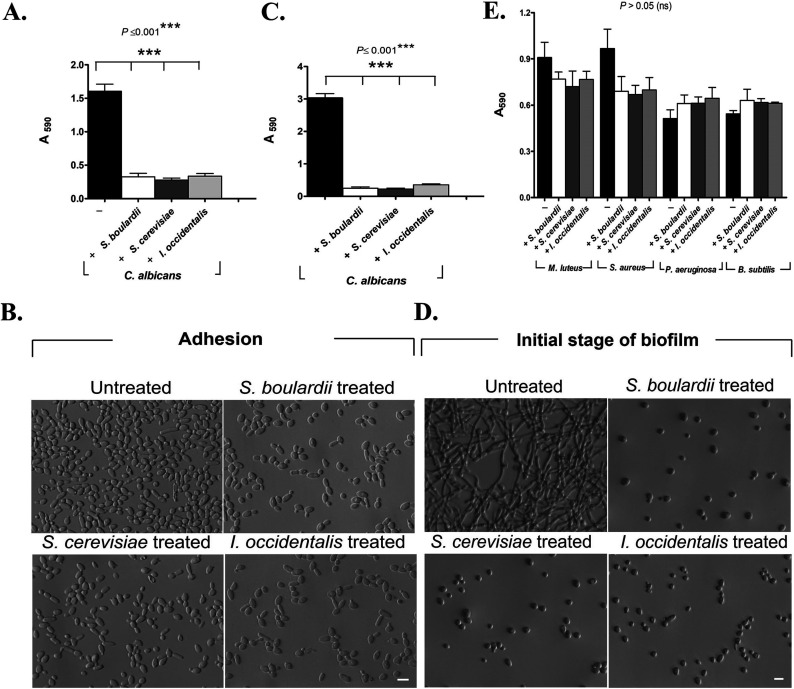
The food-derived, beneficial yeasts S. cerevisiae and I. occidentalis inhibit adhesion and biofilm formation of the opportunistic fungal pathogen C. albicans but not bacterial species. S. boulardii was used as a reference control for the purpose of comparison. (A) For adhesion inhibition, each yeast strain was coincubated (represented as “+”) with C. albicans for 120 min at 37°C in SC medium. Crystal violet (0.5%) was used to quantify the adhesion of C. albicans on plastic surface by measuring absorbance at 590 nm (*n* = 5, independent experiment runs). (B) Representative images of adhesion experiment (described above for panel A) showing C. albicans that remain attached to an abiotic surface when treated with beneficial yeasts S. cerevisiae, *I. occidentalis*, and *S. boulardii*. (C) C. albicans was coinoculated with the beneficial yeasts for 24 h at 37°C in RPMI 1640 medium to test their effect on initial biofilm formation (*n* = 8, independent experiment runs). Crystal violet (0.5%) was used to quantify the biofilm of C. albicans by measuring absorbance at 590 nm. (D) Representative images of adhesion experiment (described above for panel C) showing C. albicans inhibit biofilm formation when treated with beneficial yeasts S. cerevisiae, *I. occidentalis*, and *S. boulardii*. (E) Bacterial biofilms were treated with the beneficial yeasts S. cerevisiae and *I. occidentalis* and reference yeast *S. boulardii* in the coinoculation condition. After incubation, biofilm was quantified by crystal violet staining (*n* = 3, independent experiment runs). Values are shown as means plus standard deviations (SD) (error bars), and all values were represented at a significance with respect to the control using one-way ANOVA followed by *post hoc* analysis using Tukey’s *t* test. Bars, 5 μm. ns, not significant.

Adhesion and filamentation are the primary determinants for C. albicans virulence. C. albicans must first adhere to abiotic surfaces and then filament to form a three-dimensional biofilm. *In vitro* adhesion, filamentation, and biofilm formation assays were used to test the effect of the two food-derived yeasts Saccharomyces cerevisiae and Issatchenkia occidentalis (16) on C. albicans virulence. The beneficial yeast cell density 10^8^/ml was empirically determined as effective against adhesion of Candida albicans (strain SC5314) (14). Adhesion to abiotic surfaces was tested under three different conditions detailed in Materials and Methods. Surfaces were treated with the beneficial yeast prior to inoculating C. albicans (preinoculation condition) or were inoculated at the same time (coinoculation condition) as C. albicans. We also tested conditions where C. albicans was allowed to attach to the abiotic surface before exposure to beneficial yeast (postinoculation condition). Cells were incubated for 120 min for each of the three conditions. Our results indicate that the food-derived beneficial yeast S. cerevisiae and I. occidentalis inhibit adhesion of C. albicans to abiotic surfaces under all conditions tested (Fig. 1A and B; see also Fig. S1 in the supplemental material). Furthermore, the extent of inhibition observed with the beneficial yeast was comparable to the commercially available beneficial yeast Saccharomyces cerevisiae var. *boulardii* (S. boulardii), which has showed ∼85%, ∼60%, and ∼38% in preinoculation, coinoculation, and postinoculation condition, respectively (Fig. 1A and Fig. S1).

10.1128/mBio.01891-21.2 FIG S1 Novel, food-derived yeast strains S. cerevisiae and *I. occidentalis* after preinoculation (–) and postinoculation (→) with C. albicans in SC medium. Crystal violet (0.5%) was used for quantifying the adhered C. albicans cells on abiotic surfaces. *S. boulardii* was used as a reference condition for the purpose of comparison (*n* = 8 assay replicates). Values are means ± standard deviations (SD) (error bars), and all values were represented at a significance with respect to control using Tukey’s *t* test. Download FIG S1, JPG file, 0.09 MB. Copyright © 2021 Kunyeit et al. 2021 Kunyeit et al. https://creativecommons.org/licenses/by/4.0/ This content is distributed under the terms of the Creative Commons Attribution 4.0 International license.

Filamentation of C. albicans is an important virulence factor for deep tissue invasion, escape from immune cells, and biofilm formation (17). Beneficial yeasts S. cerevisiae and I. occidentalis inhibit filamentation of C. albicans (Fig. 1C and D) to the same extent as the commercially available beneficial yeast strain S. boulardii. We investigated the effects of beneficial yeasts on C. albicans biofilm in three distinct stages of biofilm development: initial (2 to 10 h), intermediate (24 h), and mature (48 h). Beneficial yeasts were able to inhibit biofilm formation significantly (∼79%; *P* < 0.05) on coinoculated (Fig. 1C and D) and preformed C. albicans biofilms (up to 5- to 6-h-old biofilms, under postinoculation conditions) (Fig. S2A and B). However, intermediate (24 h) and mature biofilm (48 h) did not show any reduction in biomass but exhibited a marked decrease in metabolic activity (Fig. S3A and B).

10.1128/mBio.01891-21.3 FIG S2 Effects of novel, food-derived beneficial yeasts S. cerevisiae and *I. occidentalis* on biofilms. (A) Effect on preformed, initial-stage C. albicans biofilms. C. albicans was incubated for 4 to 5 h and then treated with beneficial yeasts for 24 h. Biofilm was quantified using 0.5% crystal violet staining. (B) Micrographs showing early stages of C. albicans biofilm formation on abiotic surfaces treated with the beneficial yeasts S. cerevisiae and *I. occidentalis* and compared to the reference strain *S. boulardii* (*n* = 8 assay replicates). All values represent means ± standard deviations (SD). Significance levels were expressed with respect to control using Tukey’s *t* test. Scale bar, 10 μm. Download FIG S2, JPG file, 0.1 MB. Copyright © 2021 Kunyeit et al. 2021 Kunyeit et al. https://creativecommons.org/licenses/by/4.0/ This content is distributed under the terms of the Creative Commons Attribution 4.0 International license.

10.1128/mBio.01891-21.4 FIG S3 Effects of novel, food-derived beneficial yeasts S. cerevisiae and *I. occidentalis* on biofilms. Exposure of the beneficial yeasts S. cerevisiae and *I. occidentalis* or the reference strain *S. boulardii* to 24-h-old C. albicans biofilms did not show any reduction in biomass (A) but exhibited a marked decrease in metabolic activity (*n* = 4 assay replicates) (B). C. albicans was incubated for 24 h at 37°C. Unattached cells were removed and treated with beneficial yeasts for an additional 24 h. Biofilm formation and metabolic activity were quantified by crystal violet and MTT assay method, respectively, after a wash with PBS. ns, nonsignificant. Download FIG S3, JPG file, 0.08 MB. Copyright © 2021 Kunyeit et al. 2021 Kunyeit et al. https://creativecommons.org/licenses/by/4.0/ This content is distributed under the terms of the Creative Commons Attribution 4.0 International license.

To evaluate the effects of these novel yeasts on bacterial biofilms, we tested Micrococcus luteus, Staphylococcus aureus, Pseudomonas aeruginosa, and Bacillus subtilis. We found that beneficial yeast treatment did not inhibit bacterial biofilm (Fig. 1E), suggesting that these yeasts are not general microbial antagonists but rather exhibit specific antivirulence activity against C. albicans and non-*albicans Candida* species (14).

### Beneficial yeasts prevent attachment and morphological transition of C. albicans to human epithelial cells.

**FIG 2:**
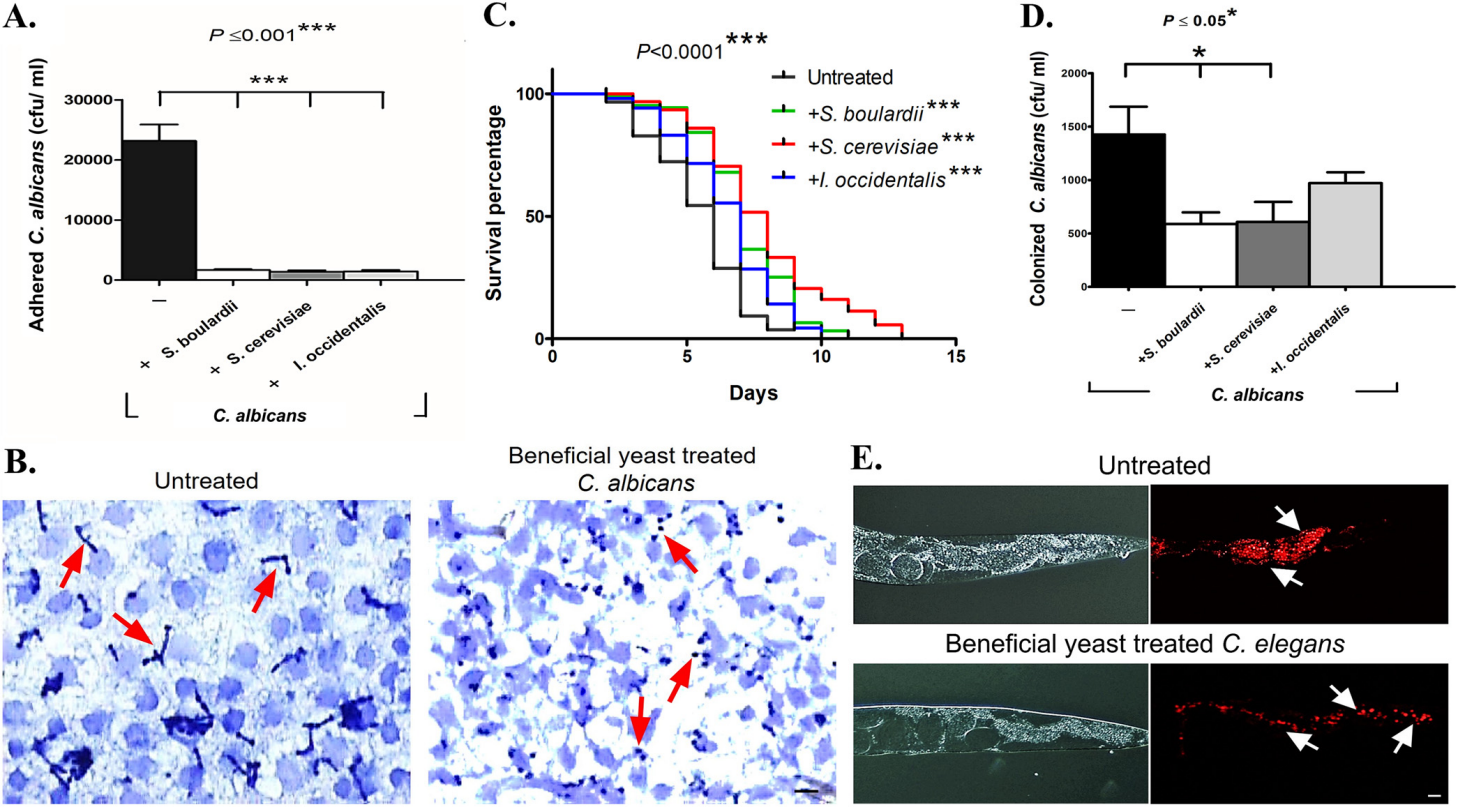
The beneficial yeasts prevent adhesion of epithelial cells and protect a live animal from C. albicans infection and colonization. (A) The novel yeasts S. cerevisiae and *I. occidentalis* were coinoculated with C. albicans on Caco-2 monolayer. Adhesion of C. albicans to the epithelial layer was quantified by assessing CFU on candida chrome agar (*n* = 3 assay replicates). (B) Microscopic images of Caco-2 monolayers treated with beneficial yeast S. cerevisiae (right) show C. albicans in ovoid yeast form cells compared to untreated controls (left) where C. albicans germ tubes are visible, indicating inhibition of morphological transition. (C) C. elegans was used as a live animal host. The life span of nematodes reared on a mixed diet of C. albicans and beneficial yeasts was plotted and compared to those reared on a diet of C. albicans alone (*n* = 4 experimental replicates, 143 ± 15 nematodes). (D) Coexposure to beneficial yeasts, S. cerevisiae, *I. occidentalis*, and *S. boulardii* reduced C. albicans colonization of the nematode gut. Colonization was quantified by counting colonies formed on candida chrome agar. Values were expressed as CFU/ml (*n* = 3). (E) Microscopic observation of colonization of nematode intestine using mCherry-tagged C. albicans. Infected C. elegans treated with S. cerevisiae reduced C. albicans colonization of the nematode intestine. Values are means plus standard deviations (SD) (error bars). Kaplan-Meier statistical analysis tools by log rank (Mantel-Cox) tests were used for the C. elegans survival assay and statistical significance is expressed with respect to control. Bar, 10 μm.

We used Caco-2 cell lines, derived from human colon, to assess the ability of the beneficial yeasts to protect the epithelial cell from attachment and morphological transition of C. albicans. We measured inhibition under three experimental conditions, preinoculation, coinoculation, and postinoculation, that mimic *in vitro* conditions tested. We assayed the ability of the putative beneficial yeasts S. cerevisiae and I. occidentalis to inhibit adhesion of C. albicans to live epithelial cells compared to untreated samples that were not exposed to beneficial yeasts. We used the commercially available beneficial yeast S. boulardii as a reference strain. Our results indicate that the beneficial yeasts were able to inhibit adhesion of C. albicans to cultured epithelial cells *ex vivo* under all conditions tested (Fig. 2A and Fig. S4). While preinoculation conditions practically abolished adhesion of C. albicans to Caco-2 monolayers, coinoculation and postinoculation conditions significantly inhibited adhesion (94% and 84%, respectively; *P* < 0.05). Furthermore, beneficial yeast treatment inhibited the morphological transition of C. albicans on Caco-2 cell monolayer (Fig. 2B). Together, these results suggest that the presence of beneficial yeasts prevents attachment of C. albicans to abiotic surfaces as well as live cells. Beneficial yeasts also inhibit filamentation of C. albicans, which likely prevents biofilm formation on abiotic surfaces and invasion of live epithelial cells.

10.1128/mBio.01891-21.5 REVISED FIG S4 C. albicans that was treated with beneficial yeasts in preinoculated (represented as –) (A) and postinoculated (represented as →) (B) conditions showed reduced attachment to Caco-2, a human epithelial cell line grown in monolayers (*n* = 3 assay replicates). Download REVISED FIG S4, JPG file, 1.5 MB [ORIGINAL FIG S4, JPG file, 0.05 MB]. Copyright © 2021 Kunyeit et al. 2021 Kunyeit et al. https://creativecommons.org/licenses/by/4.0/ This content is distributed under the terms of the Creative Commons Attribution 4.0 International license.

To test whether beneficial yeasts are able to inhibit adhesion and filamentation of C. albicans in the presence of gastric juices, we simulated the gut environment *in vitro* using gastrointestinal components, including pepsin, bile, pancreatic enzymes, electrolytes, ionic constituents, and carbohydrates with a pH of 2.5 (gastric juice) and 8.5 (bile juices) (18). We found that beneficial yeasts were able to inhibit adhesion and germ tube development of C. albicans which attached to the abiotic surfaces (Fig. S5).

10.1128/mBio.01891-21.6 FIG S5 Beneficial yeasts inhibit adhesion of C. albicans in simulated bile juice (*n* = 4 assay replicates). Download FIG S5, JPG file, 0.05 MB. Copyright © 2021 Kunyeit et al. 2021 Kunyeit et al. https://creativecommons.org/licenses/by/4.0/ This content is distributed under the terms of the Creative Commons Attribution 4.0 International license.

### Beneficial yeasts protect nematodes from C. albicans infections.

C. elegans is an ideal experimental host system to study microbial interactions in the gastrointestinal tract. The nematode gut faithfully recapitulates a mammalian intestine in anatomy, innate immunity, and neuronal circuits (19, 20). Nematodes are reared on bacteria that form their gut microbiome, thereby allowing the investigator to manipulate the intestinal microbiome (21). We leveraged these unique characteristics of C. elegans to study how the novel beneficial yeasts S. cerevisiae and I. occidentalis interact with the pathogenic fungus C. albicans in the context of a live animal intestine. To mimic the experimental conditions used for *in vitro* and *ex vivo* studies, we tested three exposure conditions: first, where worms were preexposed to beneficial yeasts prior to infection with C. albicans (preexposure); next, where worms were exposed to beneficial yeasts and C. albicans simultaneously (coexposure); and finally, where C. elegans were treated with beneficial yeasts after infection with C. albicans (postinfection). Our results indicate that C. elegans that were exposed to beneficial yeasts were better able to cope with a C. albicans infection under all conditions tested (Fig. 2C and Fig. S6A and B). Exposing worms to C. albicans and beneficial yeast at the same time (coexposure) extended host survival (by ∼3 days) compared to the infected control group. We observed that worms treated with the beneficial yeasts of the *Saccharomyces* species, S. boulardii or S. cerevisiae survived significantly longer than those treated with I. occidentalis in the coexposure condition (Fig. 2C). Worms that were exposed to beneficial yeasts prior to infection with C. albicans (preexposure) or were treated with beneficial yeasts 3 days after infection (postinfection) with C. albicans survived longer compared to untreated worms (Fig. S6A and B). Together, these results indicate that application of beneficial yeasts at any point during the course of infection confers protective function, and C. albicans-infected worms are able to recover when beneficial yeasts are applied after C. albicans has presumably colonized the intestine. Therefore, we tested the hypothesis that beneficial yeasts are able to inhibit colonization of worm gut with C. albicans. We used mCherry-tagged C. albicans strain (22) for microscopic observation of gut colonization and coupled that with CFU on a differential growth medium, *Candida* chrome agar. Our results indicate that intestinal colonization is reduced upon treatment with beneficial yeasts. Coexposure of S. cerevisiae or S. boulardii significantly reduced colonization of C. albicans in C. elegans compared to C. albicans-infected C. elegans worms that were not treated with beneficial yeasts. Treatment with the non-*Saccharomyces* beneficial yeast I. occidentalis also reduced gut colonization, but to a lesser extent (Fig. 2D and E). The postinfection condition abolished intestinal colonization of C. albicans by day 4 of beneficial yeast treatment. Preexposure reduced C. albicans colonization during first 2 days, but in subsequent days the pathogen successfully recolonized. Together, these studies suggest that beneficial yeast treatment alleviates colonization of the gut with C. albicans and although it can provide limited protection prior to an infection, beneficial yeast treatment is most effective when it is present during an active infection.

10.1128/mBio.01891-21.7 REVISED FIG S6 Effect of beneficial yeast treatment on live animals infected with C. albicans. Lifespan of C. elegans infected with C. albicans that were pretreated with beneficial yeasts (*n* = 3, 108 ± 35 nematodes) (A) and treated after C. albicans infection (*n* = 3 assay replicates, 121 ± 13 nematodes) (B). Kaplan-Meier statistical analysis tools by log rank (Mantel-Cox) tests were used for the C. elegans survival assay. ns, nonsignificant. Download REVISED FIG S6, JPG file, 1.10 MB [ORIGINAL FIG S6, JPG file, 0.08 MB]. Copyright © 2021 Kunyeit et al. 2021 Kunyeit et al. https://creativecommons.org/licenses/by/4.0/ This content is distributed under the terms of the Creative Commons Attribution 4.0 International license.

### Secretome of beneficial yeast confer protective activity.

**FIG 3:**
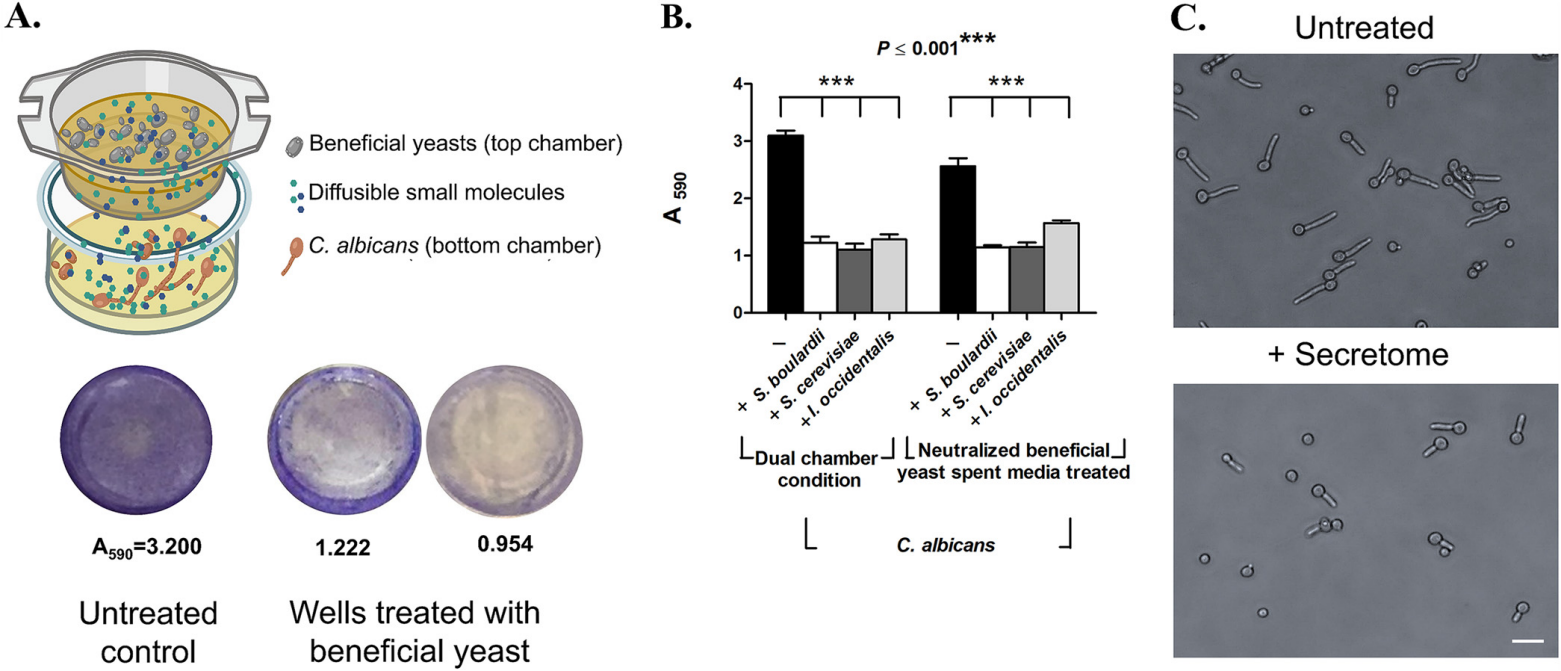
Diffusible metabolites from beneficial yeasts inhibit filamentation of C. albicans. (A) Two-chamber cell insert wells were used; these wells allow diffusion of small molecules between the two chambers. Beneficial yeasts were inoculated in the upper chamber and C. albicans in the lower chamber in SC medium. After incubation, the C. albicans-inoculated lower chamber was washed three times with PBS and stained with 0.5% crystal violet. (B) Quantification of C. albicans biomass using crystal violet staining (experiment described above for panel A, *n* = 4 assay replicates). Secretome from beneficial yeasts was exposed to C. albicans for 24 h in dual-chamber cell insert well (left bars). Neutralized secretome (right bars) was used to confirm that inhibitory effect was independent of pH changes. (C) Representative micrograph of C. albicans germ tube development after treatment (bottom) with the secretome from beneficial yeasts compared to untreated control (top). Bar, 10 μm.

To understand the mechanism of beneficial yeasts, we hypothesized that the beneficial yeasts either pose a physical barrier by occluding the intestinal epithelium or secrete small secondary metabolites that mediate beneficial yeast activity. To test whether the protective activity was retained in the secretome, we exposed C. albicans to the secretome of S. cerevisiae, I. occidentalis, and S. boulardii using a two-chamber cell culture insert (Fig. 3A and Fig. S7A). Individual live beneficial yeast strains were maintained in the upper chamber and C. albicans in the lower chamber of cell culture insert wells so that cell-to-cell contact was prevented but small molecules present in the beneficial yeast secretome were able to diffuse to the lower chamber. We observed that live beneficial yeasts S. cerevisiae, *I. occidentalis*, and *S. boulardii* effectively reduced the virulence of C. albicans (Fig. 3).

10.1128/mBio.01891-21.8 REVISED FIG S7 Effect of heat-killed beneficial yeast on C. albicans. (A) Heat-killed beneficial yeasts S. cerevisiae, *I. occidentalis*, and *S. boulardii* and live C. albicans were inoculated into the upper chamber and lower chamber of the dual-cell insert, respectively (*n* = 4, assay replicates). (B) After incubation, the lower C. albicans-inoculated chamber was washed three times with PBS and stained with 0.5% crystal violet. (C) Experimental setup to address whether the beneficial yeasts posed a physical barrier. (D) Heat-killed beneficial yeasts and live C. albicans were coinoculated and quantified by 0.5% crystal violet (*n* = 8 assay replicates). (E) A representative image showing that heat-inactivated yeast reduces adhesion but not filamentation of C. albicans. Scale bar, 10 μm. ns, nonsignificant. Download REVISED FIG S7, JPG file, 0.66 MB [ORIGINAL FIG S7, JPG file, 0.08 MB]. Copyright © 2021 Kunyeit et al. 2021 Kunyeit et al. https://creativecommons.org/licenses/by/4.0/ This content is distributed under the terms of the Creative Commons Attribution 4.0 International license.

Next, we tested whether these food-derived beneficial yeasts pose a physical barrier that inhibits adhesion of C. albicans. We introduced metabolically inactive, heat-killed beneficial yeast along with C. albicans in 96-well plates. We observed that the heat-killed beneficial yeast partially inhibit adhesion due to physical barrier effect, but C. albicans cells are able to filament and develop biofilms (Fig. S7C, D, and E). We further confirmed this finding using a contactless, dual-chamber cell culture experimental setup, where the secretome of heat-killed beneficial yeasts placed in the upper chamber was unable to inhibit the virulence of C. albicans (Fig. S7B). This is in contrast to live beneficial yeasts, which showed a robust inhibition of adhesion (Fig. 3B) and hyphal development (Fig. 3C) of C. albicans. Together, these results demonstrate that the protective activity of beneficial yeasts is primarily retained in the secretome and the barrier function offers minimal protection.

To confirm that this inhibitory effect was due to a secreted small molecule and not acidification of growth media that is known to inhibit hyphal development in C. albicans, we neutralized the spent media derived from S. cerevisiae, I. occidentalis, and S. boulardii and tested the ability of these conditioned media to inhibit filamentation of C. albicans. Our results indicate that neutralized spent media also inhibited adhesion and filamentation of C. albicans (Fig. 3B). Together, these results suggest that secondary metabolites secreted by the beneficial yeasts, and not the acidic nature of the spent media, inhibited adhesion and filamentation of C. albicans. Simultaneously, we tested whether the secretome of beneficial yeasts was able to kill C. albicans. We used an agar diffusion assay and found that exposure to the secretome of beneficial yeast did not reduce viability of C. albicans (Fig. S8).

10.1128/mBio.01891-21.9 FIG S8 Agar diffusion assay results indicate that putative probiotic yeasts S. cerevisiae (strain KTP) (c), *I. occidentalis* (ApC) (g), and reference probiotic strain *S. boulardii* (a) do not inhibit C. albicans compared to ketoconazole (10 μg) used as positive control which showed a clear zone of growth inhibitory around the disc (j). Other yeast strains were also used in this assay. Download FIG S8, JPG file, 0.1 MB. Copyright © 2021 Kunyeit et al. 2021 Kunyeit et al. https://creativecommons.org/licenses/by/4.0/ This content is distributed under the terms of the Creative Commons Attribution 4.0 International license.

**FIG 4:**
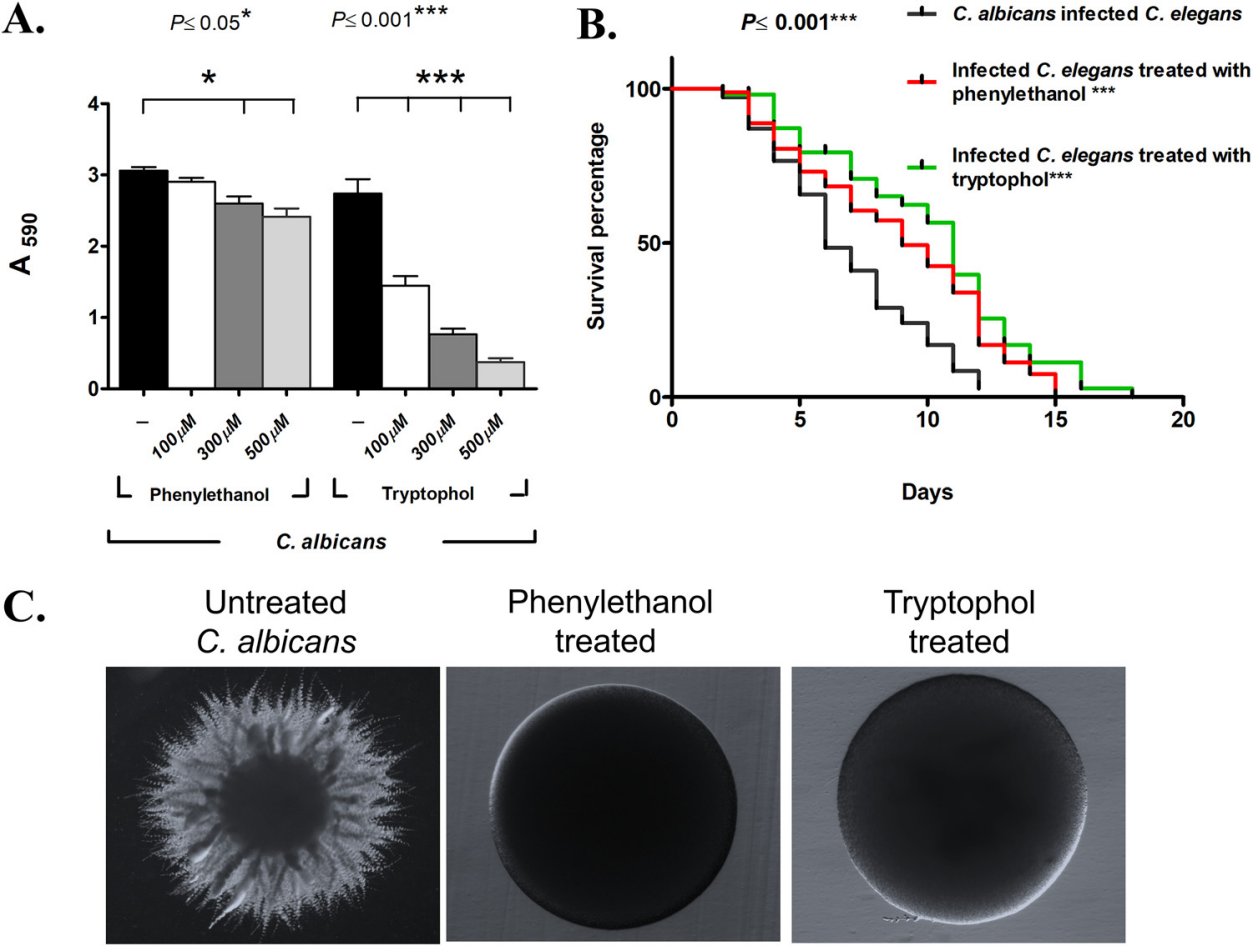
Phenylethanol and tryptophol are sufficient for beneficial activity *in vitro* and *in vivo.* (A) Various concentrations (100, 300, and 500 μM) of commercially procured aromatic alcohols phenylethanol and tryptophol were exposed to C. albicans, and biomass was measured at 590 nm after crystal violet staining (*n* = 5 experimental replicates). (B) Life span of C. elegans infected with C. albicans that are treated with tryptophol (100 μM) (green curve) and phenylethanol (100 μM) (red curve) (*n* = 3 experimental replicates, 82 ± 28 nematodes). (C) Representative images showing filamentation of C. albicans upon phenylethanol and tryptophol treatment. C. albicans was inoculated into spider agar medium supplemented with aromatic alcohols, tryptophol and phenylethanol. Images were captured on a dissection microscope after 4 days of incubation. Values are means plus standard deviations (SD) (error bars). Kaplan-Meier statistical analysis tools by log rank (Mantel-Cox) tests were used for the C. elegans survival assay.

We and others have observed that beneficial yeast action is dose dependent, where cell density (10^8^/ml) plays a key role in adhesion and hyphal inhibitory ability of *Candida* species (14, 23). Furthermore, heat-killed beneficial yeasts were largely ineffective (Fig. S7A and B). Therefore, we tested the hypothesis that secondary metabolites, responsive to cell density, inhibit virulence of C. albicans. We used liquid chromatography followed by tandem mass spectroscopy (LC-MS/MS) to separate and analyze the mass of the predicted secondary metabolites in the spent media. We confirmed the presence of two aromatic alcohols, tryptophol and phenylethanol, secreted by several yeast species (7, 24) that have recently been shown to inhibit C. albicans filamentation (7, 25). High-pressure liquid chromatography (HPLC) quantification revealed that the secretome of the beneficial yeasts S. cerevisiae, I. occidentalis, and S. boulardii contained 140 to 150 μM phenylethanol and 120 to 178 μM tryptophol (Table 1 and Fig. S9). Next, we tested whether pure tryptophol or phenylethanol was able to inhibit virulence of C. albicans and confer host protection of C. elegans against C. albicans infection. We tested commercially available purified tryptophol and phenylethanol and confirmed that these compounds inhibited filamentation of C. albicans (Fig. 4C). We found that tryptophol and phenylethanol effectively inhibited adhesion and filamentation (Fig. 4A), as well as protected C. elegans from C. albicans infection (Fig. 4B). Together, these results indicate that the secondary metabolites tryptophol and phenylethanol are sufficient for the beneficial activity against C. albicans.

**TABLE 1:** Aromatic alcohol concentration of yeasts used in the study

Yeast species or strain	Phenylethanol (μM)	Tryptophol (μM)
*S. boulardii*	154 ± 11.43	168 ± 06.27
S. cerevisiae	153 ± 14.18	120 ± 04.07
*I. occidentalis*	143 ± 07.36	178 ± 01.36
S. cerevisiae (wild type)	170 ± 18.03	164 ± 07.67
S. cerevisiae *aro8 aro9* mutant	51 ± 05.91	22 ± 04.89

10.1128/mBio.01891-21.10 FIG S9 Representative high-pressure liquid chromatograms (HPLC) of phenylethanol and tryptophol of standards (A), beneficial yeast secretome (B), and S. cerevisiae mutant *aro8 aro9* that is defective in phenylethanol and tryptophol production (C). Download FIG S9, JPG file, 0.1 MB. Copyright © 2021 Kunyeit et al. 2021 Kunyeit et al. https://creativecommons.org/licenses/by/4.0/ This content is distributed under the terms of the Creative Commons Attribution 4.0 International license.

### Mutants that do not produce the secondary metabolites are ineffective.

**FIG 5:**
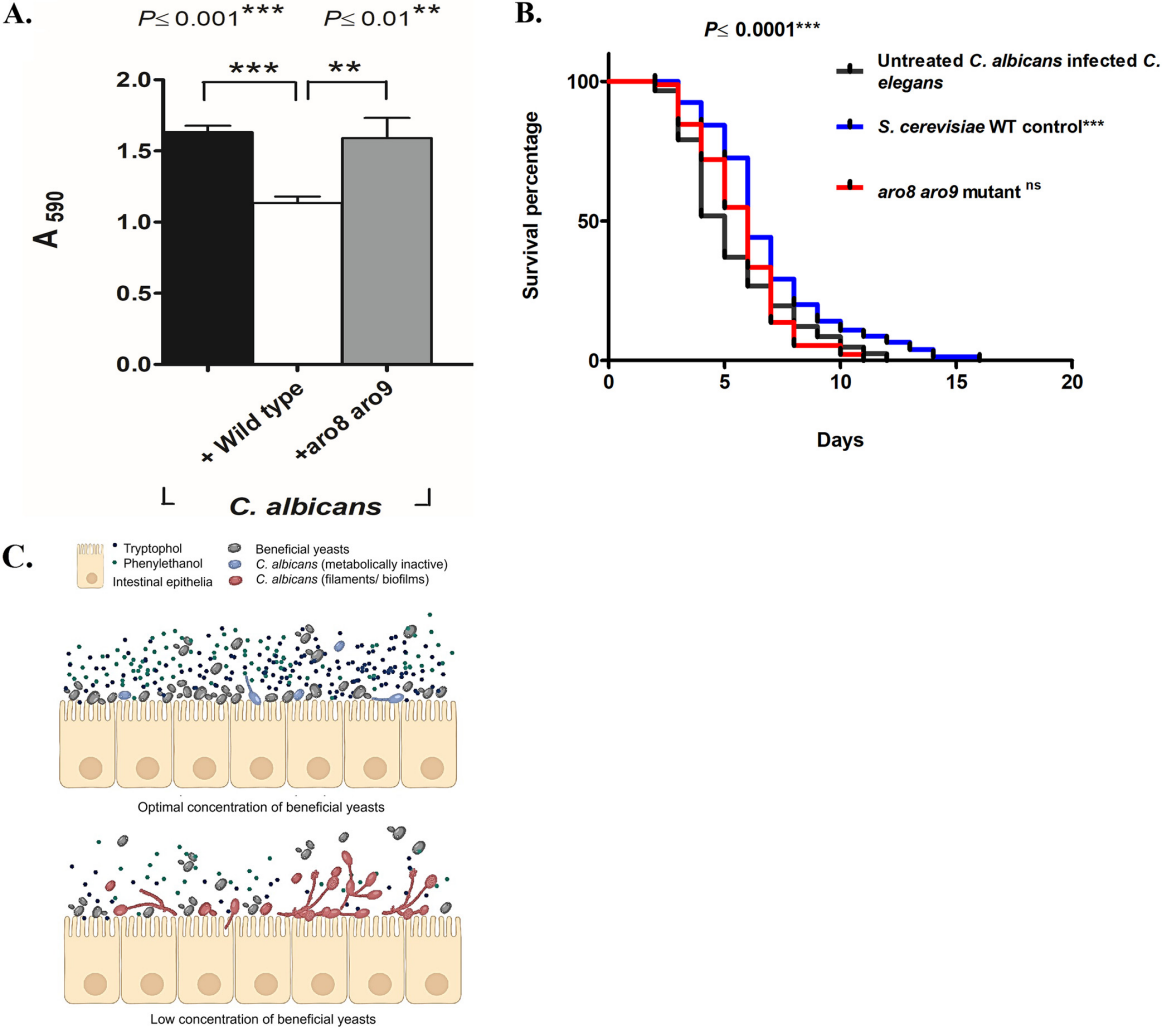
S. cerevisiae *aro8 aro9* mutant analysis shows phenylethanol and tryptophol are required for beneficial activity *in vitro* and *in vivo.* (A) The neutralized secretome of the *aro8 aro9* double mutant (gray bar) that does not produce aromatic alcohols was treated for its ability to prevent biofilm of C. albicans for 10 h at 37°C compared to its isogenic wild-type counterpart (white bar) or untreated controls (black bars) (*n* = 4 assay replicates). (B) Life span of C. elegans tested either with *aro8 aro9* double mutant or its wild-type (WT) counterpart. The double mutant (red curve) does not protect the nematode as compared to its isogenic wild-type control strain (blue curve) (*n* = 4 experimental replicates, 163 ± 11 nematodes). (C) Working model for the mechanism by which beneficial yeasts function to inhibit C. albicans virulence. The cartoon depicts that beneficial yeast at an optimal cell density (top row) protects the intestinal tract by secreting bioactive metabolites, tryptophol and phenylethanol but not at lower cell density (bottom row). Values are means plus standard deviations (SD) (error bars). Kaplan-Meier statistical analysis tools by log rank (Mantel-Cox) tests were used for the C. elegans survival assay, and statistical significance is expressed with respect to control. ns, nonsignificant.

To test the hypothesis that yeast-derived aromatic alcohols are necessary for protection against C. albicans, we compared a laboratory strain of S. cerevisiae (F1322 [see Table S1 in the supplemental material]) and the isogenic *aro8 aro9* double mutant (L8184), which is unable to produce these aromatic alcohols (7). We used neutralized spent media to test the ability of the secretome from the *aro8 aro9* mutant to inhibit adhesion and filamentation of C. albicans compared to the isogenic wild-type control. Our results indicate that wild-type yeast inhibits virulence of C. albicans compared to the isogenic *aro8 aro9* double mutant that is unable to produce tryptophol and phenylethanol (Fig. 5A). To confirm these results *in vivo*, we coinfected C. elegans with C. albicans and either the wild-type control strain or the *aro8 aro9* mutant strain. Our results demonstrate that nematodes coinfected with wild-type S. cerevisiae survived longer than those that were coinfected with the *aro8 aro9* mutant (Fig. 5B), suggesting that S. cerevisiae produces aromatic alcohols that inhibit virulence traits of C. albicans, thereby protecting the nematode host from C. albicans infection. Together, these provide a molecular mechanism for the action of food-derived beneficial yeasts where the diffusible secondary metabolites tryptophol and phenylethanol contained in their secretome are largely responsible for their protective properties against the opportunistic pathogenic fungi C. albicans (Fig. 5C). This report demonstrates that tryptophol and phenylethanol are necessary and sufficient for the beneficial activity of the novel food-derived yeasts.

10.1128/mBio.01891-21.1 TABLE S1 Yeast and bacterial strains used in the study. Download Table S1, PDF file, 0.03 MB. Copyright © 2021 Kunyeit et al. 2021 Kunyeit et al. https://creativecommons.org/licenses/by/4.0/ This content is distributed under the terms of the Creative Commons Attribution 4.0 International license.

## DISCUSSION

Morphological transition of Candida albicans is a major virulence factor (26) that is associated with biofilm development on implanted medical devices and host epithelium and that helps the pathogen evade the immune system (4, 5). Previous studies have reported beneficial activity of microbes (11, 27, 28). Other disparate studies have shown that secondary metabolites harbor antimicrobial properties (29, 30). This report links these disparate studies to demonstrate that the probiotic activity of beneficial microbes is mediated primarily by secondary metabolites they secrete. This study finally connects the findings of various elegant studies by using a simple, yet relevant host model—C. elegans, that probes the contribution of host innate immunity without the “noise” of other aspects of host immunity. The innate immunity of C. elegans has been conserved through evolution; therefore, information gained in this study is relevant in mammals. Invertebrate animal studies such as this, with large sample sizes (>100 nematodes), are required to justify mammalian studies where sample size is restricted (typically 5). In this study, we demonstrate that food-derived beneficial yeasts S. cerevisiae and I. occidentalis, at 10^8^ cells/ml, inhibit C. albicans virulence and infection. This cell density is in line with the prescribed dosage of commercially available probiotic yeast S. boulardii, where 10^7^ to 10^9^ CFU/day has been shown to be effective in inhibiting C. albicans in murine models as well as in humans (13, 31). Prior *in vitro* studies of beneficial microbe activity against various infections, including candidiasis, have been recapitulated in preclinical models and clinical settings (11, 32). Systematic preclinical studies using murine models are substantiated by these findings in metazoans and will determine the dosage of food-derived yeasts S. cerevisiae and I. occidentalis.

Mature biofilms are highly resistant to various therapeutic agents. Commercial antifungals such as fluconazole, caspofungin, and amphotericin B reduce metabolic activity (33). While application of the putative beneficial yeasts did not decrease biomass of mature biofilms, they significantly reduced (*P* < 0.05) metabolic activity of the biofilm. Our results are in line with prior reports (34) where bioactive metabolites derived from S. boulardii were also ineffective against mature biofilm (12). Application of cell-free, neutralized spent media from beneficial yeasts also reduced the metabolic activity of 24-h-old C. albicans biofilm by 26%, suggesting that soluble metabolites play a crucial role in controlling *Candida* growth. It has been suggested that reduced viability, rather than complete elimination of the biomass and filamentation, may be a better avenue for keeping *Candida* in its commensal state. This decrease in metabolic activity has been proposed as a mechanism for the beneficial effect of Lactobacillus rhamnosus (35).

Yeast produces aromatic alcohols such as tryptophol and phenylethanol in a cell density-dependent manner (7). They produce 140 to 200 μM tryptophol and phenylethanol (36) at a cell density of >10^7^/ml. At high cell density, C. albicans likely accumulates aromatic alcohols and inhibits filamentation (10). However, such high cell count is likely not achieved under physiological conditions, since colonization of the host gut with C. albicans would adversely affect the host. Food-derived beneficial yeasts, however, are able to attain a higher cell density in the host gut. To mimic these conditions, we used 10^5^ to 10^6^ cells/ml of C. albicans and 10^8^ cells/ml of beneficial yeasts. Under these conditions, the concentrations of aromatic alcohols produced by C. albicans (20 to 35 μM) are negligible compared to those produced by beneficial yeasts (140 to 150 μM phenylethanol and 120 to 170 μM tryptophol). These concentrations of aromatic alcohols tryptophol and phenylethanol inhibit the C. albicans filamentation and reduce biomass. Previous reports have shown that intraperitoneal administration of phenylethanol (70 μM) and other C. albicans autoregulatory alcohols have protected mice from invasive candidiasis (37). Similarly, pretreatment of tryptophol (200 μM) in C. albicans-infected Galleria mellonella larvae (greater wax moth or honeycomb moth larvae) enhanced survival by 88.89% compared to untreated C. albicans-infected larvae (38). The conditions of the gastrointestinal tract have been shown to be favorable for the production of aromatic alcohols by C. albicans (39). In line with published reports, our observations in simulated gastrointestinal tract conditions indicate that treatment with beneficial yeast inhibits *Candida* virulence. The gastrointestinal tract is a complex environment where beneficial microbes are typically confined to the gut, where they have a local effect. However, the diffusible aromatic alcohols they produce are able to mount a systemic and long-ranging response.

Overall, using *in vitro*, *ex vivo*, and *in vivo* readouts, this study demonstrates that food-derived beneficial yeasts produce aromatic alcohols, specifically tryptophol and phenylethanol, that inhibit virulence of C. albicans. Since heat-inactivated beneficial yeasts retain minimal activity, we propose that beneficial yeasts present a chemical inhibition as well as pose a physical (passive) barrier that prevent *Candida* infections. Therefore, they may be used as an alternative to, or in combination with, traditional antifungal drugs.

## MATERIALS AND METHODS

### Microbial strains, medium preparation, and chemicals used.

Bacterial and yeast strains were maintained in Luria broth (LB) and yeast extract-peptone-dextrose (YPD), respectively, unless otherwise mentioned (see Table S1 in the supplemental material). Synthetic complete (SC) and RPMI 1640 media were used to study adhesion and biofilm experiments, respectively. Pepsin, ox bile, pancreatin Dulbecco’s modified Eagle medium (DMEM), and fetal bovine serum (FBS) were procured from HiMedia Laboratories Pvt. Ltd. RPMI 1640, yeast synthetic drop-out medium supplements, yeast nitrogen base, tryptophol, phenylethanol, and 3-(4,5-dimethylthiazol-2-yl)-2,5-diphenyltetrazolium bromide (MTT) were obtained from Sigma-Aldrich.

Saccharomyces cerevisiae (strain KTP; deposition number, NCIM 3672) and Issatchenkia occidentalis (ApC, NCIM 3668) were cultured in YPD medium for 18 h at 28°C. Cells were collected and used for the assays which are described below. Saccharomyces cerevisiae var. *boulardii* (S. boulardii), used as a reference yeast strain in the study, was procured from National Collection of Dairy Cultures (NCDC)-NDRI, India (NCDC 363).

Spider medium was used to monitor filamentation of C. albicans on agar plates. Briefly, C. albicans was spotted on spider agar plate containing 300, 500, and 1,000 μM tryptophol and phenylethanol and incubated for 4 days at 37°C. Images were captured on a Zeiss dissection microscope equipped with SPOT imaging software. To monitor the effects of aromatic alcohols phenylethanol and tryptophol on C. albicans in liquid media, C. albicans (10^6^/ml) was inoculated in SC medium containing a range of (100 to 500 μM) of phenylethanol or tryptophol and incubated at 37°C for 8 to 10 h. Adhesion of C. albicans was quantified as previously described (40). Briefly, the unattached cells were removed, and plates were air dried and incubated with 50 μl of 0.5% crystal violet for 25 min. Adherent cells were washed with phosphate-buffered saline (PBS), destained in 95% (vol/vol) ethanol, and quantified by measuring absorbance at 590 nm.

### Preparation of secretome from beneficial yeast.

To prepare the secretomes from beneficial yeasts, cultures (10^8^/ml) were maintained initially for 24 h in SC medium with 50 μM ammonium sulfate at 37°C with aeration. Cell pellets were removed by centrifugation. Depleted nutrients were replenished using a standard composition of SC medium, the pH of the spent medium was neutralized, and filter sterilized prior to testing.

### Preparation simulated gastric and bile juices.

Simulated gastric and bile juices were prepared as previously described (41). In brief, gastric juice was prepared using glucose (3.5 g/liter), sodium chloride (2.05 g/liter), potassium phosphate monobasic (0.6 g/liter), calcium chloride (0.11 g/liter), potassium chloride (0.37 g/liter), and pepsin (0.7 g/liter) with pH adjusted to 2.5. Bile juice medium contained glucose (3.5 g/liter), sodium chloride (2.05 g/liter), potassium phosphate monobasic (0.6 g/liter), pancreatin (0.750 g/liter), and bile (0.5 g/liter) with pH 8.50. The reference strain *S. boulardii* or novel yeast strains of S. cerevisiae and I. occidentalis (10^8^/ml) were coinoculated with C. albicans (10^7^/ml) and incubated for 180 min at 37°C in simulated gastric juice and bile juices. Adherent cells were quantified by crystal violet staining described above.

### *In vitro* plastic adhesion and biofilm assays.

*In vitro* assays were performed in preinoculation, coinoculation, and postinoculation conditions as previously described (14). In brief, for preinoculation experiments, the beneficial yeasts were first inoculated (10^8^/ml) into 96-well plates and incubated with SC medium for 30 min at 37°C. C. albicans cells (10^7^/ml) were then introduced and incubated for an additional 90 min. For coinoculation conditions, the beneficial yeasts and C. albicans cells were incubated together for 120 min at 37°C. For postinoculation conditions, C. albicans cells were inoculated into 96-well plates for 30 min before then, beneficial yeast was introduced on the C. albicans cells that had adhered to the bottom of the well and incubated for another 90 min. After treatment, plates were washed three times with phosphate-buffered saline (PBS) (pH 7.4) to remove unattached cells. Adherent C. albicans cells were either microscopically visualized or quantified by crystal violet staining method as described above (40, 42). Preinoculation and coinoculation conditions test the hypothesis that the beneficial yeasts are prophylactic, while the postinoculation conditions test whether beneficial yeasts are therapeutic.

We also tested the effects of beneficial yeasts on the adhesion of Staphylococcus aureus, Micrococcus luteus, Pseudomonas aeruginosa, and Bacillus subtilis. Bacterial culture (optical density of 0.5) and 10^8^/ml of beneficial yeast was maintained in SC medium and incubated for 48 h at 37°C. After incubation, unattached cells were removed, and attached bacterial cells were quantified by 0.5% crystal violet (40).

C. albicans biofilms were tested as described earlier (14) at three distinct biofilm development phases, initial (0 to 10 h), intermediate (24 h), and mature (48 h) (43). Initial stages of biofilm were tested in two ways, coinoculation treatment, where C. albicans (10^6^/ml) and beneficial yeasts (10^8^/ml) were coincubated in RPMI 1640 medium for 24 h at 37°C or postinoculation conditions, where C. albicans was allowed to attach and initiate biofilm formation for 90 min prior to introduction of 10^8^/ml beneficial yeasts. Plates were incubated 24 h at 37°C in mild shaking (90 rpm). Intermediate and mature biofilm treatments were conducted on 24- and 48-h-old preformed C. albicans biofilm, respectively. Briefly, 10^6^/ml of C. albicans was inoculated into RPMI 1640 medium for 30 min. To develop a stable biofilm, unadhered C. albicans cells were removed and incubated at 37°C for an additional 24 h or 48 h for intermediate or mature biofilms, respectively. Unattached *Candida* cells were removed from the intermediate/mature biofilm plate. Beneficial yeasts (10^8^/ml) were then introduced to these biofilms and incubated for another 24 h. Crystal violet staining was used for quantifying the biofilm of C. albicans, which is explained above.

### Assessing metabolic activity of biofilm of C. albicans.

The metabolic activity of biofilms was determined using tetrazolium dye MTT (44). Intermediate (24-h-old) and mature (48-h-old) C. albicans biofilm was treated with beneficial yeasts for 24 h. After treatment, unattached cells were removed by PBS washing. Then, 10 μl of MTT solution (5 mg/ml, stock concentration) was added to each untreated and treated 96-well plate and made up to 100 μl. The plates were incubated at 37°C for 4 h. After incubation, unreacted MTT solution was removed. Then, dimethyl sulfoxide (DMSO) was used to dissolve formazan crystals. Dissolved formazan crystals were transferred to a new 96-well plate, and absorbance was read at 540 nm.

### *Ex vivo* adhesion of C. albicans to intestinal epithelium.

Caco-2 cells, a standard human epithelial colon cell line, were used to monitor the effects of beneficial yeasts on interaction between C. albicans and intestinal epithelia. A total of 5 × 10^4^ cells/ml of Caco-2 cells were seeded into 96-well microtiter plates containing DMEM with 10% FBS and were incubated at 37°C with 5% CO_2_. The experiment was performed as described earlier (14). After 20 days, the resulting monolayer of Caco-2 cells was treated with C. albicans and beneficial yeasts. Experiments were conducted in preinoculation, coinoculation, and postinoculation conditions analogous to the plastic adhesion experiment described above.

C. albicans CFU were assessed using candida chrome agar plates. Yeast cells that remained unattached to the Caco-2 monolayer were removed by PBS washing. Cells were then detached off the surface of the culture plates using cell scrapers. The Caco-2 cells were lysed using a pestle, and CFU of attached C. albicans cells were assessed by viable count method using candida chrome agar plates. In addition, crystal violet stain (0.5%) was used to stain yeasts cells that remained adherent to the Caco-2 cell monolayer for visualization. Micrographs were taken using bright-field microscope for qualitative observation.

### *In vivo* C. elegans infection and colonization assay.

C. elegans was used as a model host to monitor the effects of beneficial yeast upon C. albicans infection (45). For this *in vivo* assay, nematode eggs were harvested from six to eight worms reared on a nematode growth medium (NGM) agar plate containing Escherichia coli OP50 at 20°C for 3 days. Forty to 50 eggs were transferred to a fresh NGM plate containing E. coli OP50. Assays were conducted in preexposure, cocultured, and postexposure conditions (14). For preexposure treatment, larval stage 3 (L3)-L4 level worms were maintained on a diet of beneficial yeasts for 2 days and then transferred to a C. albicans diet. Viability was scored daily. Worms reared on a regular diet of E. coli OP50 prior to infection with C. albicans were used as an untreated control group. For coexposure treatment, worms were raised on mixed cultures of beneficial yeasts (10^6^ cells) and C. albicans (10^6^ cells), while for postinfection treatment, worms were first infected with C. albicans (10^6^ cells, for 3 days) and subsequently transferred into a beneficial yeast lawn (10^6^ cells). C. albicans-infected worms that were treated with the beneficial yeast were compared with the control group.

Five or six worms grown in preexposure, coexposure, and/or postexposure conditions were allowed to crawl on a fresh unseeded NGM plate to remove yeast or E. coli cells that might have attached to the outer cuticle of the nematode. Washed worms were transferred to an Eppendorf tube containing 100 μl PBS, then crushed using pellet pestle, and vortexed for 3 to 4 min. Finally, viable CFU of C. albicans were counted by plating an appropriate dilution on candida chrome agar to differentiate C. albicans cells from beneficial yeast isolates. To visualize colonization of the nematode gut with C. albicans, an mCherry-tagged C. albicans was used, and images were captured using a Zeiss Axiovert 200M fluorescence microscope.

### Preparation of heat-killed beneficial yeast cells.

Beneficial yeasts (10^8^ cells/ml) were incubated at 70 to 75°C for 30 min in the water bath and then washed three times with PBS buffer. Cell death was confirmed by using methylene blue staining.

### Two-chamber cell insert assay to test the secretome from beneficial yeasts.

To assess the beneficial yeast metabolite effect on C. albicans, a dual-chamber cell insert chamber (HiMedia Laboratories Pvt. Ltd.) was used. The dual-chamber cell insert has a filter (0.4-μm pore size) which separates the beneficial yeasts from C. albicans cells. Briefly, 10^8^ cells/ml of beneficial yeasts (either live or heat-killed cells were used depending on the individual experiment) and 10^6^ cells/ml of C. albicans strain were maintained in the upper and lower chamber, respectively, and incubated for 24 h at 37°C with mild shaking (90 rpm). After incubation, the upper chamber containing beneficial yeast was discarded, and the lower part of the plate containing C. albicans was washed three times with PBS (pH 7.4) to remove unadhered C. albicans cells. Finally, adherent cells were stained with 0.5% crystal violet for quantification (14).

### HPLC and LC-MS/MS analysis of tryptophol and phenylethanol.

Supernatant from 24-h-old cultures of the two novel yeasts S. cerevisiae and I. occidentalis or the reference strain *S. boulardii* was used to quantify tryptophol and phenylethanol by high-pressure liquid chromatography (HPLC) (Shimagzu Corporation, Japan). Yeast cells (10^8^ cells/ml) were cultured in SC medium containing low ammonium sulfate (50 μM) for 24 h at 37°C. The supernatant was collected by centrifugation, filtered, and analyzed by HPLC using a C_18_ column. The column was first washed with 0.01% (vol/vol) trifluoroacetic acid (TFA) dissolved in water. Following this, 0.01% (vol/vol) TFA dissolved in acetonitrile was mixed in gradually over 12 min to 10%, raised to 75% within a 1-min time span, and then maintained at 75% for 15 min. Finally, TFA was raised from 75% to 100% within a 1-min time span and maintained at 100% for 8 min. The flow rate through the column was maintained at 1 ml/min. Aromatic alcohols were detected at 210 nm. To confirm the aromatic alcohols, hybrid quadrupole time of flight (TOF) LC-MS/MS (Sciex Triple ToF 5600, Singapore) was used with the solvent system (46).

### Statistical analysis.

Statistical significance was assessed by one-way analysis of variance (ANOVA) followed by *post hoc* analysis using Tukey’s *t* test at a significance level of *P* < 0.05. Results were expressed as means ± standard deviations (SD). Analyses were performed with GraphPad Prism 5 software (GraphPad Software Inc., San Diego, CA, USA). Kaplan-Meier statistical analysis tools were used for the C. elegans survival assay.
