# Erratum for Kunyeit et al., “Secondary Metabolites from Food-Derived Yeasts Inhibit Virulence of Candida albicans”

**DOI:** 10.1128/mBio.02828-21

**Published:** 2021-10-19

**Authors:** Lohith Kunyeit, Nawneet K. Kurrey, K. A. Anu-Appaiah, Reeta P. Rao

**Affiliations:** a Department of Microbiology and Fermentation Technology, CSIR-Central Food Technological Research Institute (CFTRI), Mysuru, India; b Academy of Scientific and Innovative Research (AcSIR), Ghaziabad, India; c Department of Biology and Biotechnology, Worcester Polytechnic Institute, Worcester, Massachusetts, USA; d Department of Biochemistry, CSIR-Central Food Technological Research Institute (CFTRI), Mysuru, India

## ERRATUM

Volume 12, no. 4, e01891-21, 2021, https://doi.org/10.1128/mBio.01891-21. The article has been updated to show the revised files for Fig. S4, S6, and S7 in the supplemental material.

