# Supplementary figures and images for: Secondary Metabolites from Food-Derived Yeasts Inhibit Virulence of Candida albicans
Source: mBio. 2021 Aug 17;12(4):e01891-21. doi: 10.1128/mBio.01891-21 (PMC8406282; doi:10.1128/mBio.01891-21)

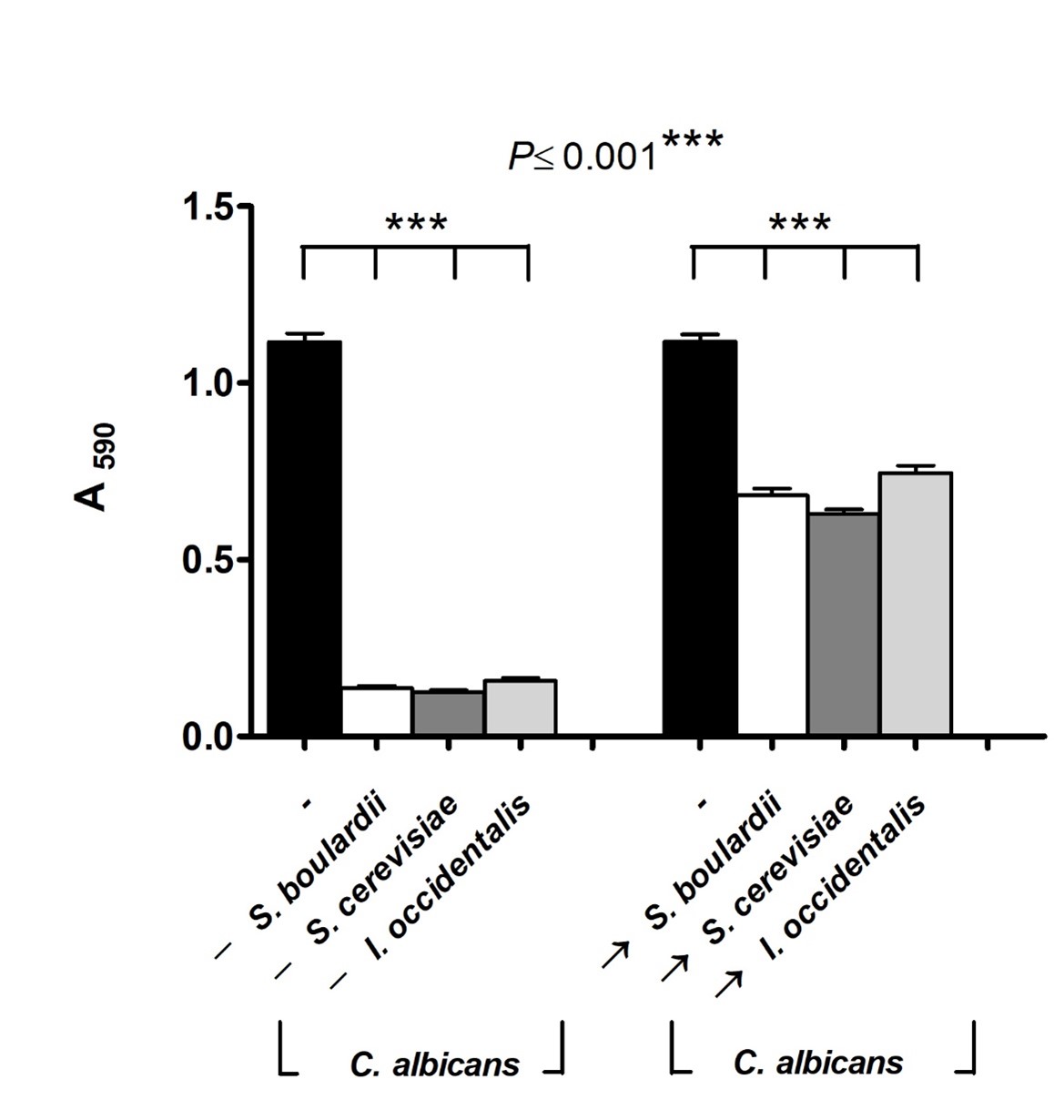

Supplement: FIG S1 [file mbio.01891-21-sf001.jpg]

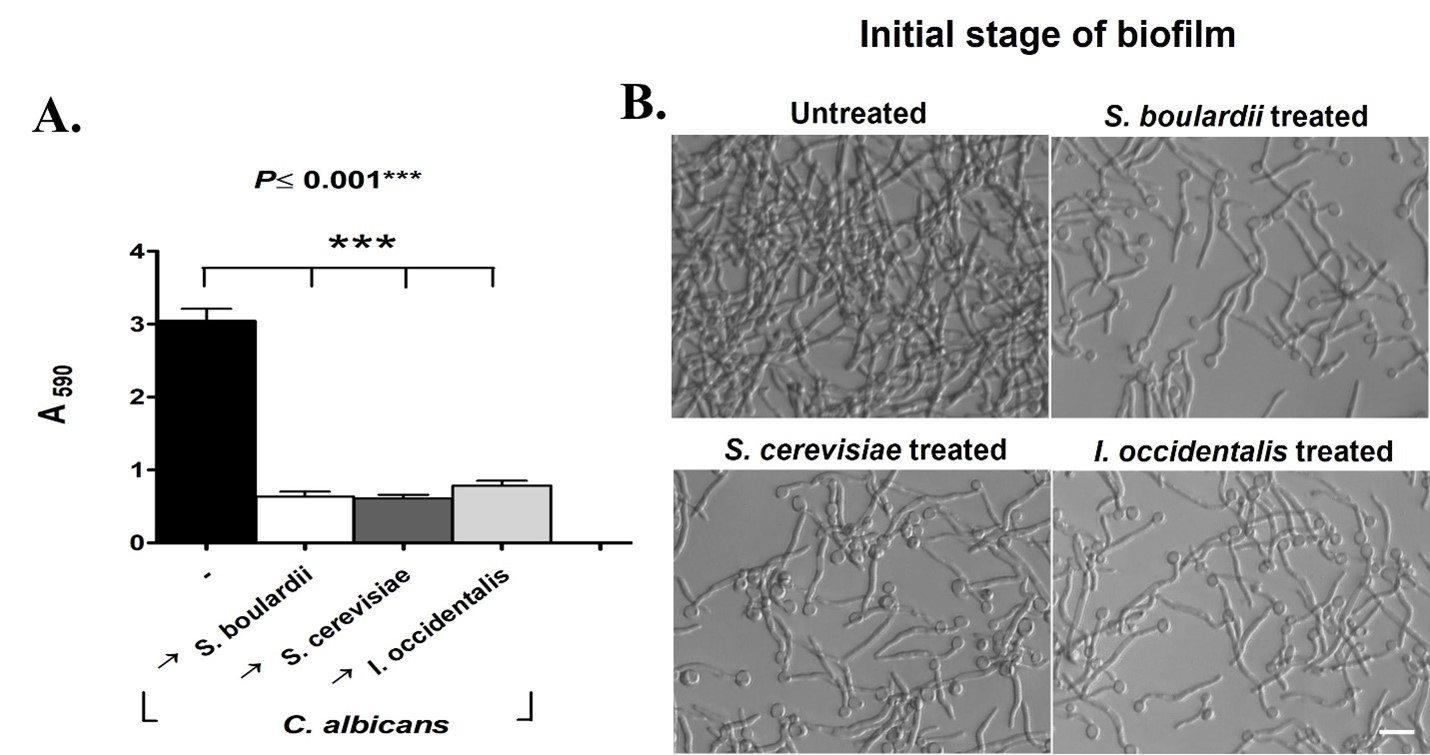

Supplement: FIG S2 [file mbio.01891-21-sf002.jpg]

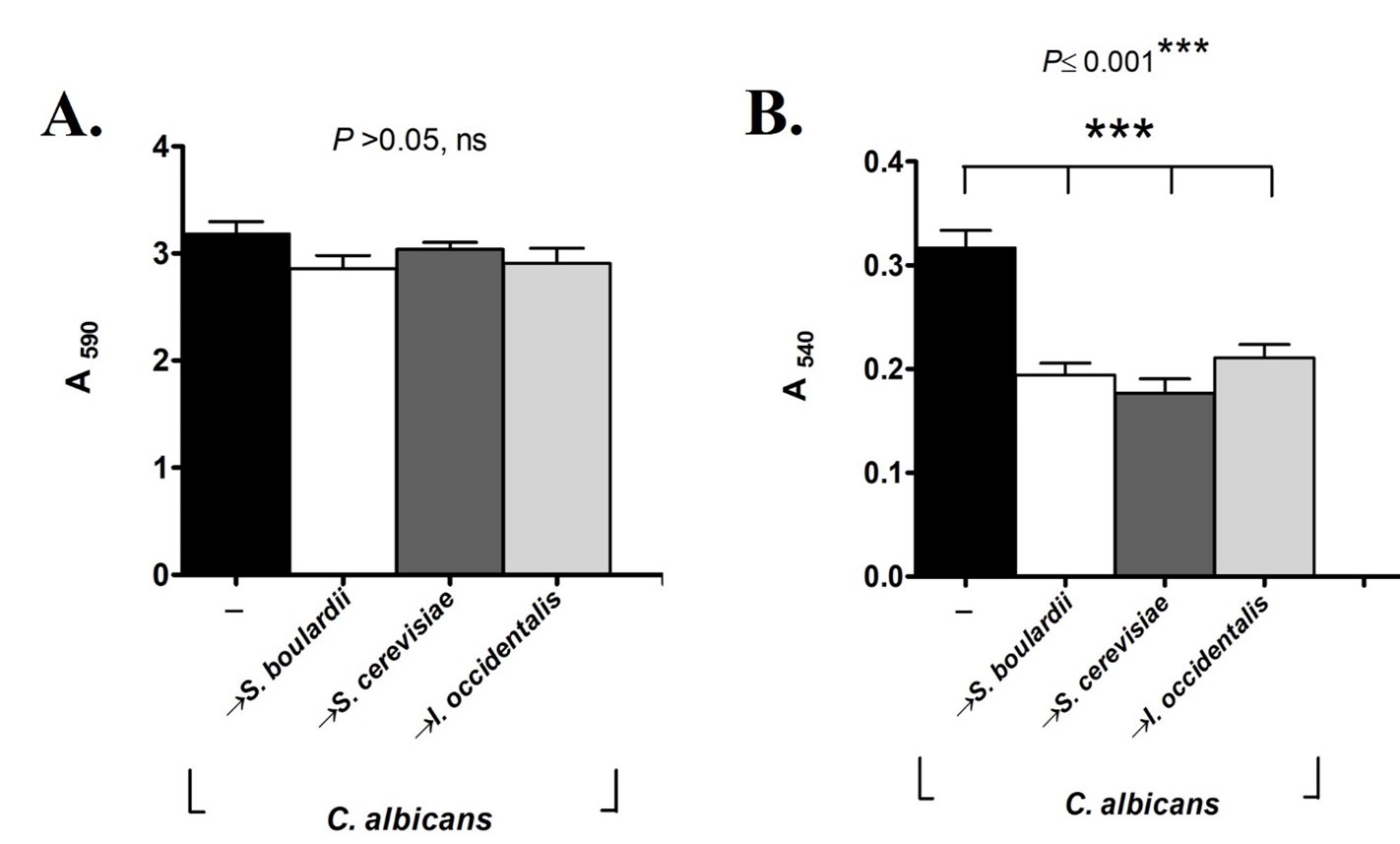

Supplement: FIG S3 [file mbio.01891-21-sf003.jpg]

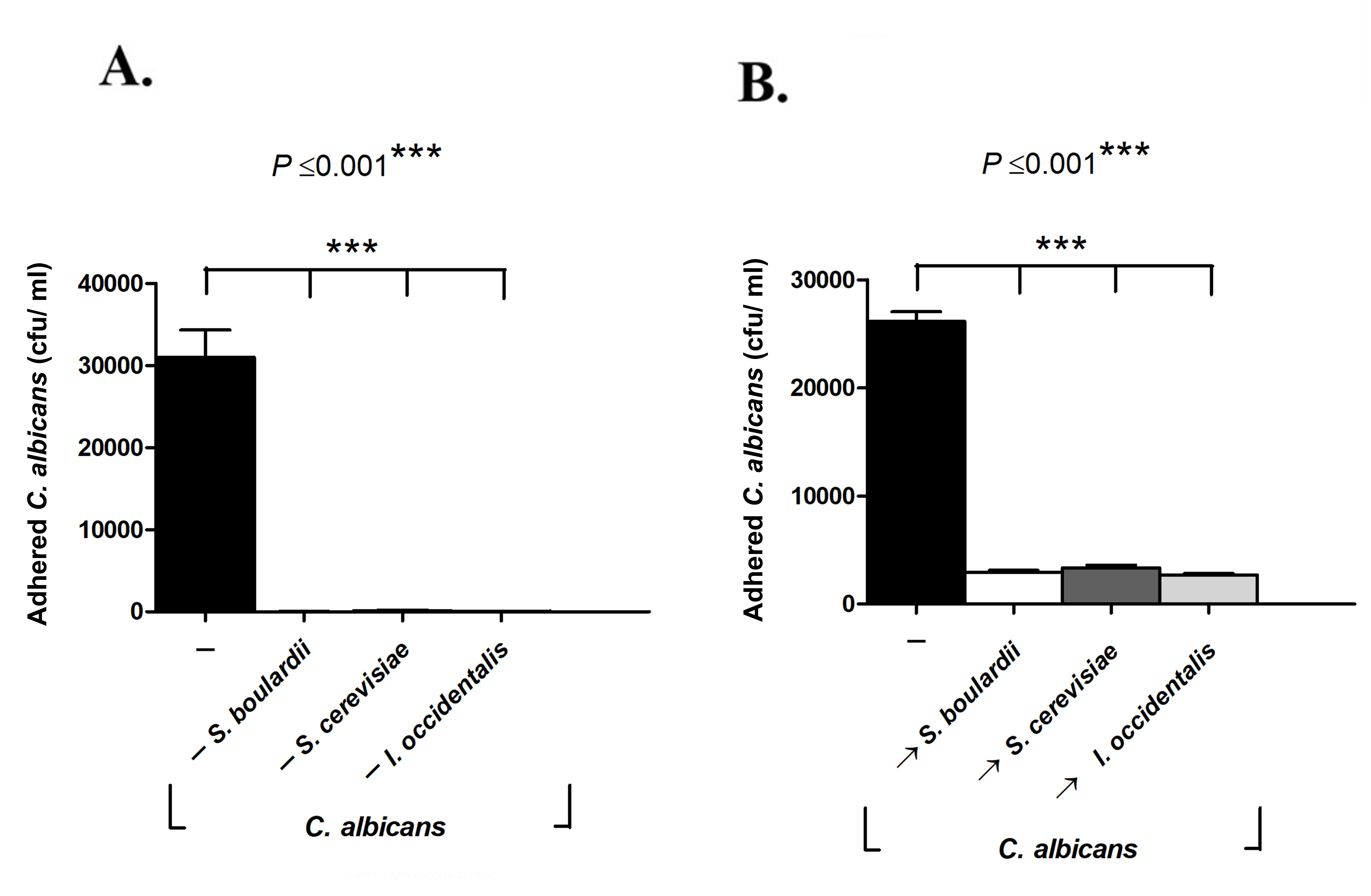

Supplement: REVISED FIG S4 [file mbio.01891-21-sf004-revised.jpg]

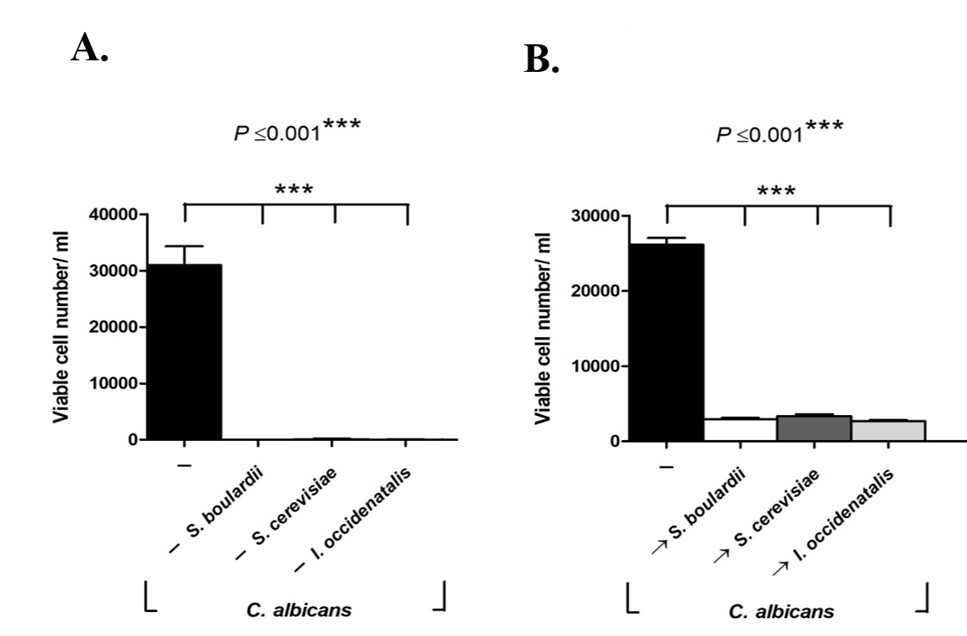

Supplement: REVISED FIG S4 [file mbio.01891-21-sf004-original.jpg]

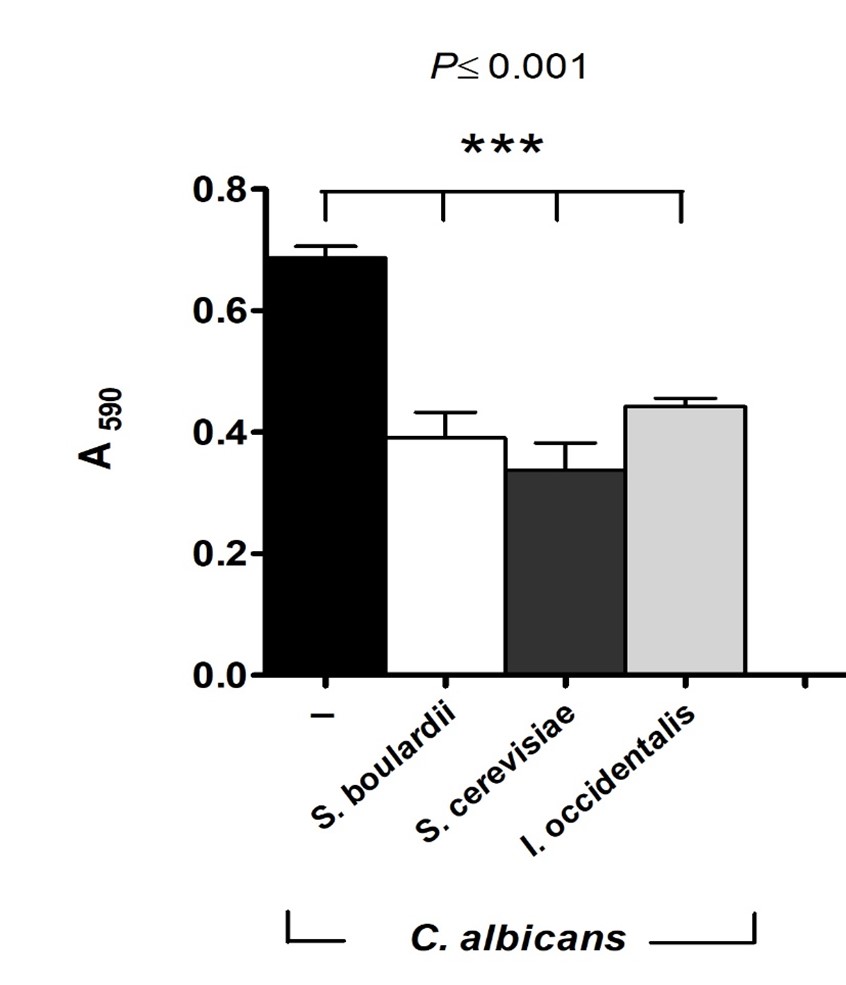

Supplement: FIG S5 [file mbio.01891-21-sf005.jpg]

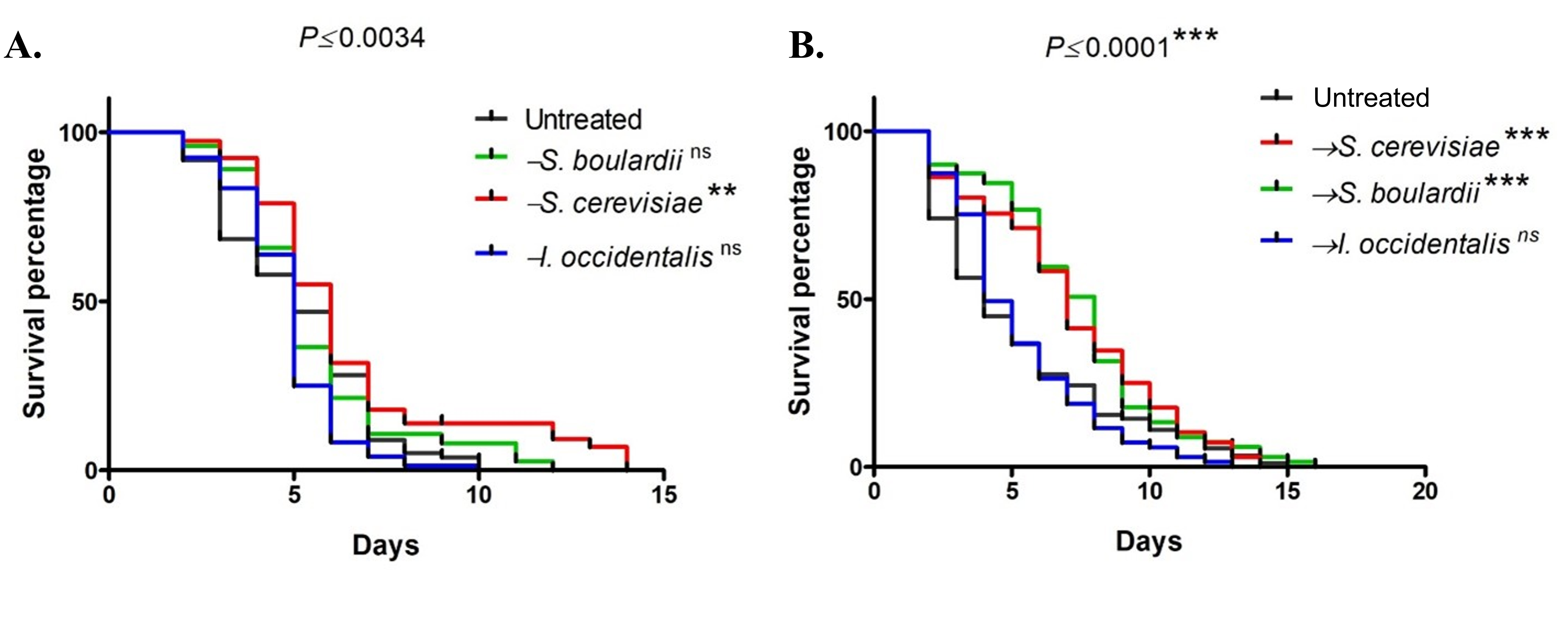

Supplement: REVISED FIG S6 [file mbio.01891-21-sf006-revised.jpg]

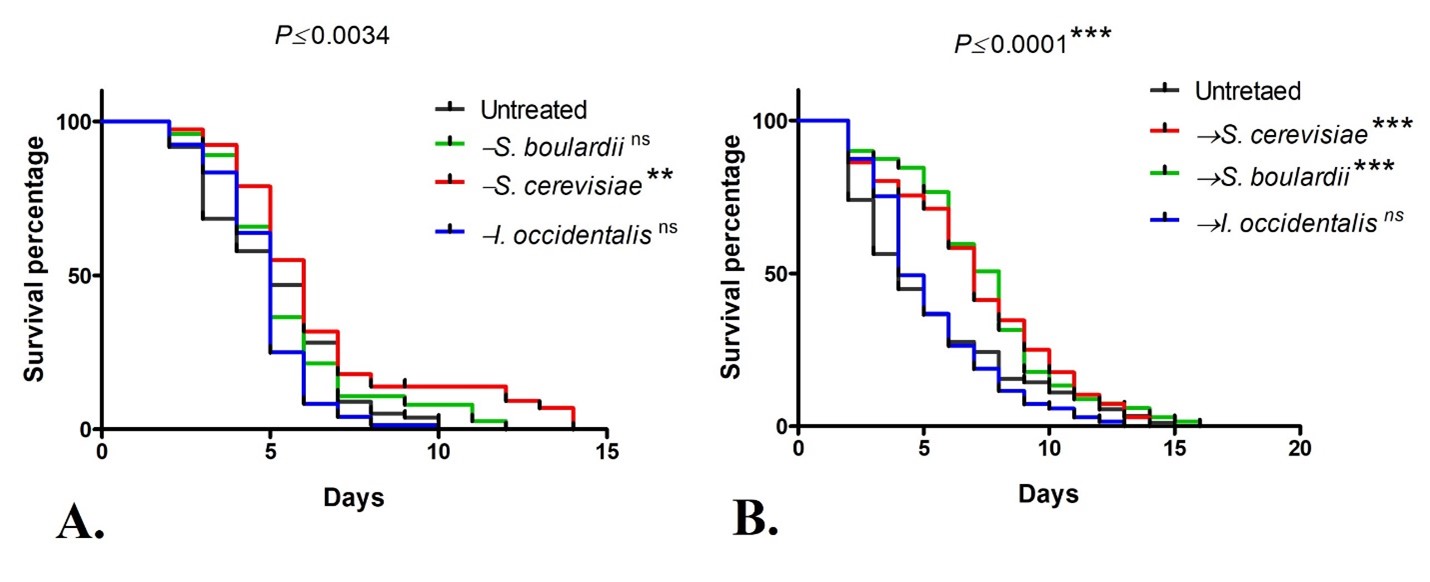

Supplement: REVISED FIG S6 [file mbio.01891-21-sf006-original.jpg]

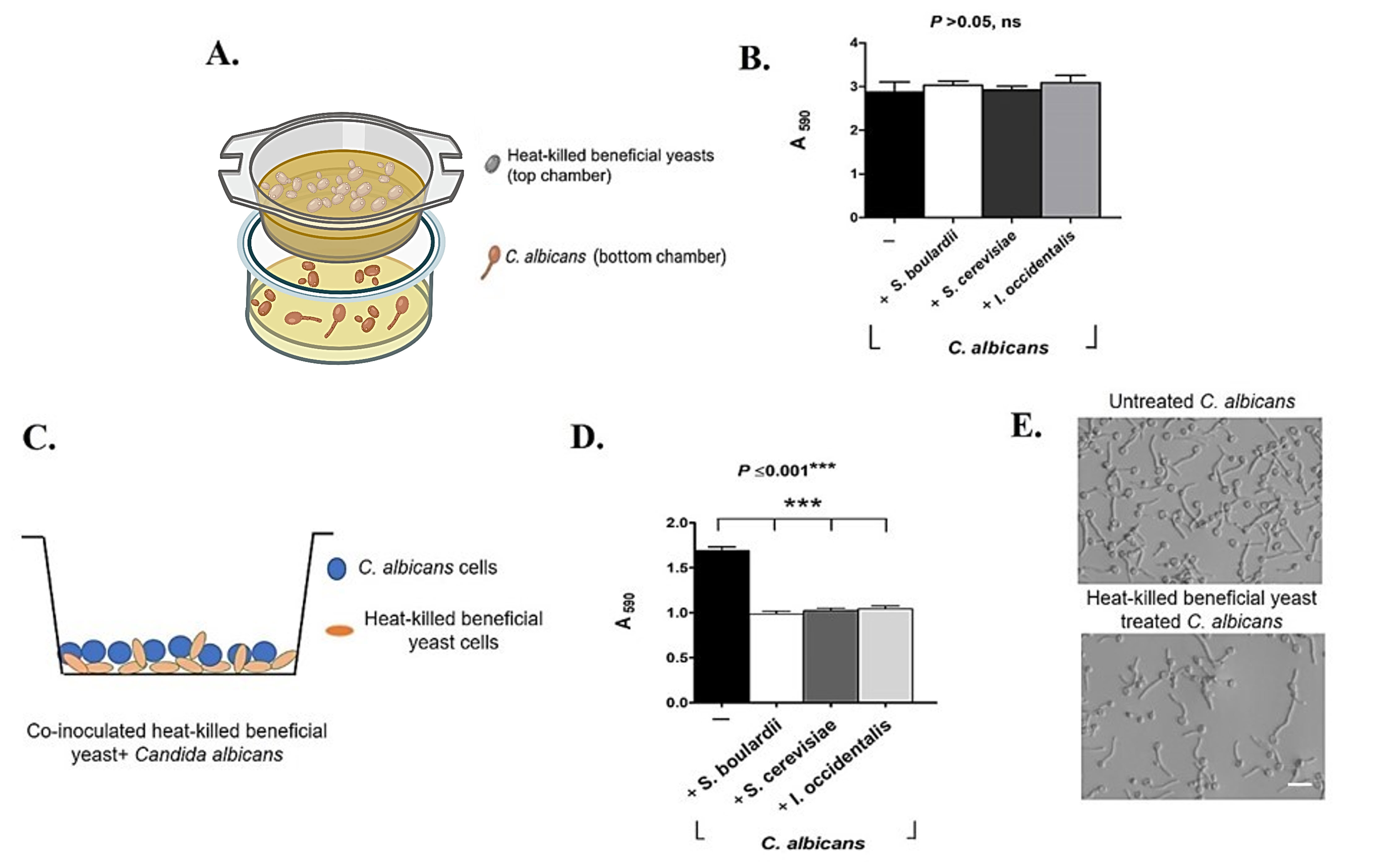

Supplement: REVISED FIG S7 [file mbio.01891-21-sf007-revised.jpg]

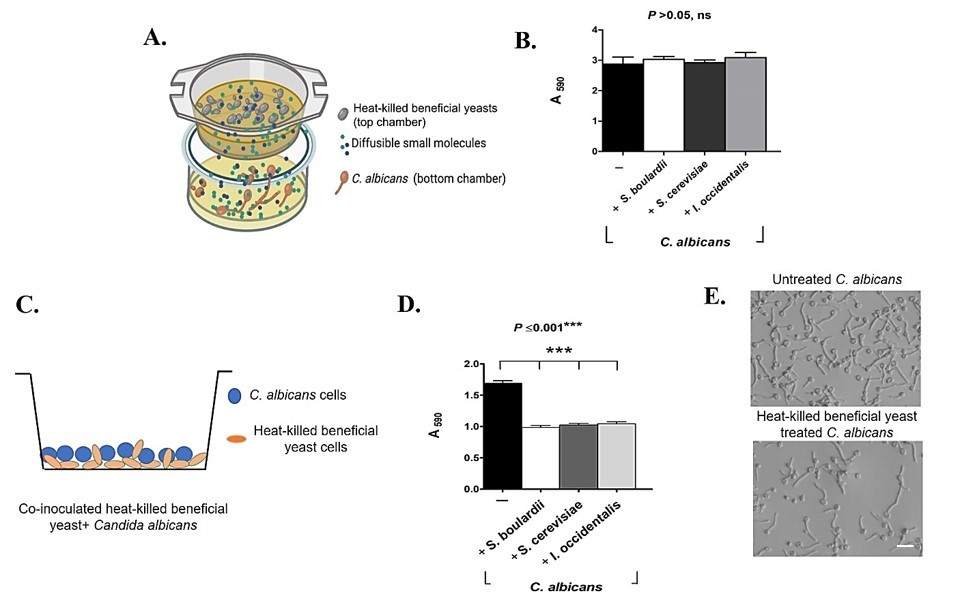

Supplement: REVISED FIG S7 [file mbio.01891-21-sf007-original.jpg]

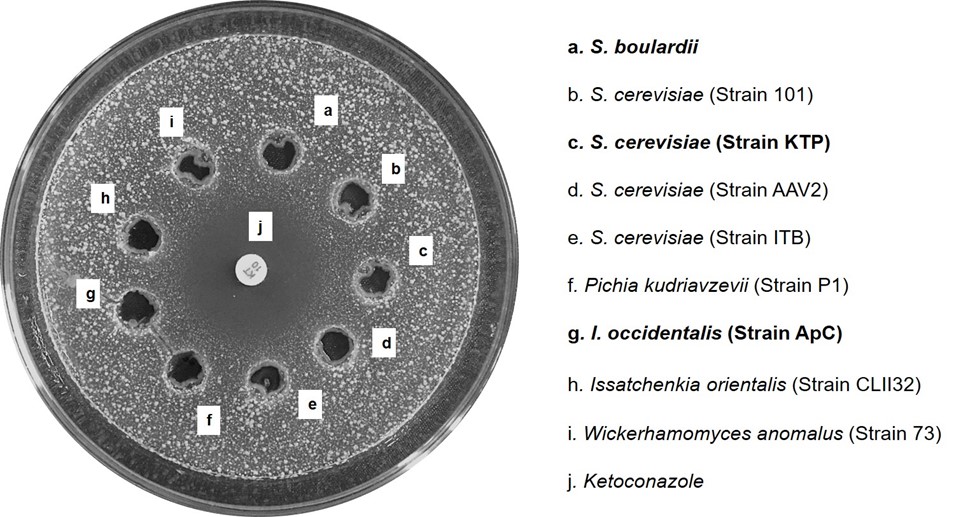

Supplement: FIG S8 [file mbio.01891-21-sf008.jpg]

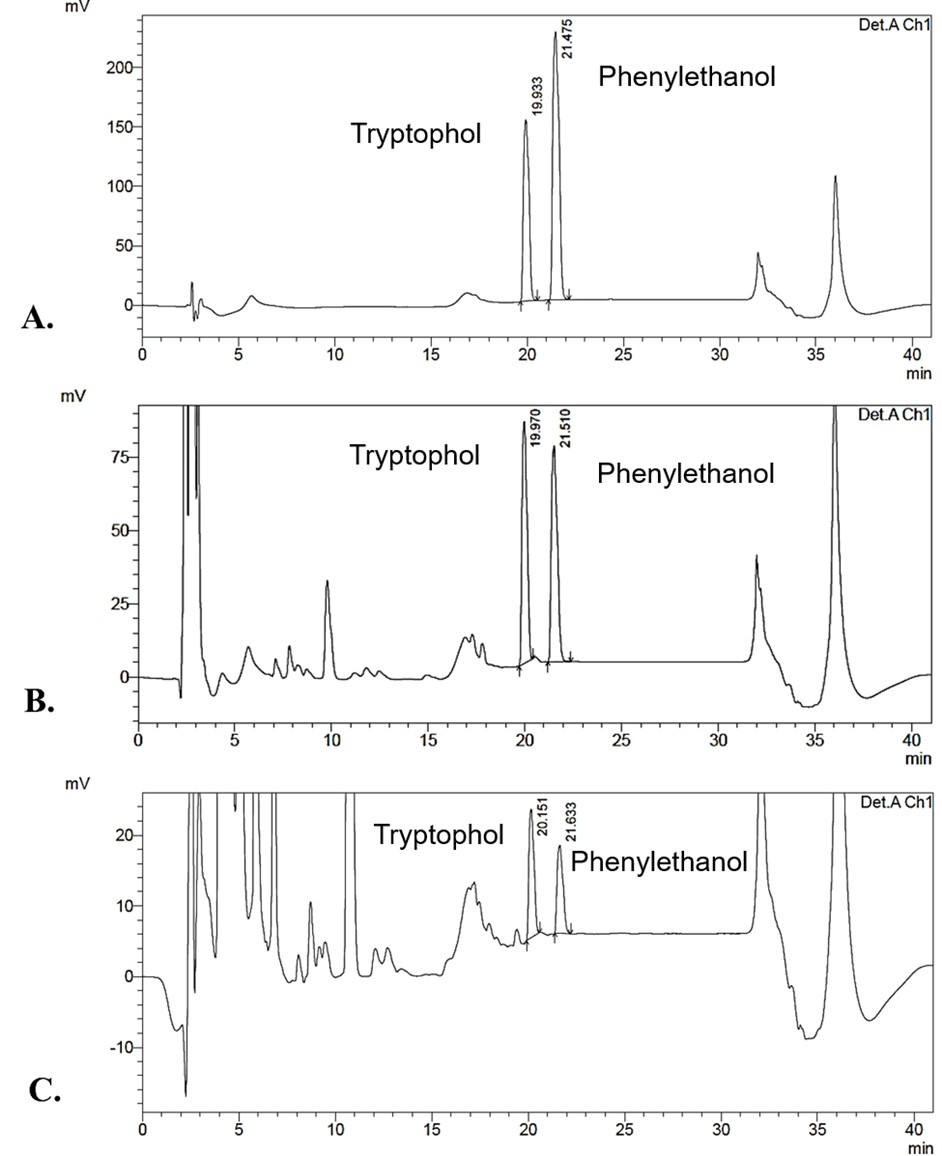

Supplement: FIG S9 [file mbio.01891-21-sf009.jpg]
