# Supplementary material for: Secondary Metabolites from Food-Derived Yeasts Inhibit Virulence of Candida albicans
Source: mBio. 2021 Aug 17;12(4):e01891-21. doi: 10.1128/mBio.01891-21 (PMC8406282; doi:10.1128/mBio.01891-21)
Supplement: TABLE S1 [file mbio.01891-21-st001.pdf]

Supplement Table 1. Yeast and bacterial strains used in the study.

**Yeasts/ bacteria**	**Strain name**	**Accession number/ deposition number**
Saccharomyces cerevisiae	KTP	NCIM 3672
Saccharomyces cerevisiae	F1322	Chen and Fink, 2006
Saccharomyces cerevisiae	L8184	Chen and Fink, 2006
Saccharomyces cerevisiae var *boulardii*		NCDC 363
Issatchenkia occidentalis	ApC	NCIM 3668
Staphylococcus aureus	-	FRI722
Micrococcus luteus	-	ATCC9341
Pseudomonas aeruginosa	-	MTCC 2297
Bacillus subtilis	-	MTCC 736
